# Defects and Strain Engineering of Structural, Elastic, and Electronic Properties of Boron-Phosphide Monolayer: A Hybrid Density Functional Theory Study

**DOI:** 10.3390/nano11061395

**Published:** 2021-05-25

**Authors:** Fang-Qiang Li, Yang Zhang, Sheng-Li Zhang

**Affiliations:** 1 School of Electronic and Information Engineering, Changshu Institute of Technology, Changshu 215500, China; lfq@cslg.edu.cn; 2 Ministry of Education Key Laboratory for Nonequilibrium Synthesis and Modulation of Condensed Matter, School of Physics, Xi’an Jiaotong University, Xi’an 710049, China; zhangsl@xjtu.edu.cn

**Keywords:** boron-phosphide monolayer, defect, strain, electronic property, first-principles study

## Abstract

Defects and in-plane strain have significant effects on the electronic properties of two-dimensional nanostructures. However, due to the influence of substrate and environmental conditions, defects and strain are inevitable during the growth or processing. In this study, hybrid density functional theory was employed to systematically investigate the electronic properties of boron-phosphide monolayers tuned by the in-plane biaxial strain and defects. Four types of defects were considered: B-vacancy (B_v), P-vacancy (P_v), double vacancy (D_v), and Stone–Wales (S-W). Charge density difference and Bader charge analysis were performed to characterize the structural properties of defective monolayers. All of these defects could result in the boron-phosphide monolayer being much softer with anisotropic in-plane Young’s modulus, which is different from the isotropic modulus of the pure layer. The calculated electronic structures show that the P_v, D_v, and S-W defective monolayers are indirect band gap semiconductors, while the B_v defective system is metallic, which is different from the direct band gap of the pure boron-phosphide monolayer. In addition, the in-plane biaxial strain can monotonically tune the band gap of the boron-phosphide monolayer. The band gap increases with the increasing tension strain, while it decreases as the compression strain increases. Our results suggest that the defects and in-plane strain are effective for tuning the electronic properties of the boron-phosphide monolayer, which could motivate further studies to exploit the promising application in electronics and optoelectronics based on the boron-phosphide monolayer.

## 1. Introduction

Two-dimensional (2D) nanostructures with an atomic thickness have attracted an increasing amount of attention due to their large surface-to-volume ratio and unique electronic properties. Graphene, a representative for 2D nanostructures, was first prepared by mechanical exfoliation (repeated peeling) of highly oriented pyrolytic graphite in 2004 [1]. Due to its ultra-high electron mobility, anomalous quantum Hall effect, ballistic transport at room temperature, and atomic thickness, graphene has been regarded as an ideal candidate material for the next generation of nanoelectronic devices [2,3,4]. Inspired by graphene, other 2D nanosheets, such as silicene [5], phosphorene [6,7], and borophene [8], have recently been experimentally synthesized. Similar to graphene, silicene is also a zero-gap semiconductor. Although both silicene and graphene have a very high Fermi velocity, the gapless features limit their direct application in nanoelectronic and nanophotonic devices. Phosphorene, a new atomically thin nanostructure with a moderate band gap, good carrier mobility, and high on/off ratio, is considered to be greatly promising for replacing silicene in the field of nanoelectronics. However, phosphorene has a fatal disadvantage in its structural stability. If exposed to visible light, phosphorene can react with water vapor and oxygen and be completely decomposed within hours [9,10]. Therefore, finding new 2D nanostructures with an appropriate band gap and good structural stability is critical for their potential applications.

Among the hexagonal honeycomb nanostructures, the boron-phosphide monolayer is a typical binary compound with good structural and mechanical stability, adjustable polarity of transport, and high carrier mobility [11,12,13], and it is expected to be experimentally synthesized. Unlike graphene and silicene, the boron-phosphide monolayer is a direct band gap semiconductor with a band gap of 0.94 eV [13,14,15]. A recent theoretical study revealed that n-type and p-type doped boron-phosphide monolayers can be used as an ideal 2D p-n junction, exhibiting diode characteristics with a large current rectification and negative differential resistance [12]. Due to the periodic affinity sites and excellent surface electrochemical properties, boron-phosphide monolayers can be expected to use as anode materials for lithium-ion batteries and anchoring materials for lithium-ion batteries [14,15]. In addition, its isomer with orthorhombic symmetry was proposed using the genetic algorithm methods [16], which holds great promise in alkali metal ion battery [17,18]. Although the boron-phosphide monolayer has not been synthesized, many studies have shown its great potential in nanoelectronics. For practical applications in electronic devices, tailoring the electronic properties is highly desirable. Generally, defects and in-plane strains are inevitable during the experimental synthesis of 2D nanomaterials because of the influence of environmental conditions and substrate, which have a very significant effect on the structural and electronic properties of 2D nanomaterials [19,20,21,22,23,24,25,26]. For example, using first-principles calculations, Wei et al. [21] explored the effect of vacancy defects on the electronic properties of the hexagonal BN monolayer and found that B_vacancy, N_vacancy, and double vacancy defects can induce direct to indirect band gap transition. Moreover, the B_vacancy or N_vacancy can also result in the magnetic properties of the BN monolayer. Hu et al. [23] found that in-plane strains can not only induce phase transition in the InSe monolayer but also trigger the indirect-to-direct band gap transition.

For the hexagonal boron-phosphide monolayer, what are the effects of defects and strain on the electronic properties? Furthermore, the influence of these factors on the structural and electronic properties plays a key role in the application of nanoelectronic devices. In this study, the first-principles study based on hybrid density functional theory was performed to systematically explore the effects of defects and strain on the structural and electronic properties of the boron-phosphide monolayer. The screened hybrid density functional HSE06 was employed to accurately evaluate the band gaps of the pure and defective boron-phosphide monolayers for exploiting their potential applications in nanoelectronics and optoelectronics.

## 2. Computational Methods

Our calculations were performed using the first-principles study based on spin-polarized density functional theory (DFT) within the projector augmented plane wave method, as implemented in Vienna ab initio simulation package (VASP) [27,28]. The generalized gradient approximation (GGA) with the Perdew–Burke–Ernzerhof (PBE) functional was employed to describe the electron exchange–correlation interaction [29,30]. The kinetic energy cutoff of the plane-wave basis expansion and the convergence of total energy were set to 450 and 10^−5^ eV, respectively. The integration of the Brillouin zone was sampled by using a Gamma-centered *k*-point grid. The selection of *k*-points was optimized with the energy error of the system smaller than 1 meV/BP. All of the studied boron-phosphide monolayers, including the pure, defective, and strained ones, were modeled in the *x*–*y* plane. For the pure and strained layers, a hexagonal primitive cell was adopted. Thus, the optimized *k*-points grid was 17 × 17 × 1. For the defective systems, an orthogonal 4 × 3 supercell containing 4 × 4 primitive cells was used, and the optimized *k*-points grid was 5 × 5 × 1. To eliminate the interactions between neighbor layers, a vacuum space of 15 Å was applied along the normal direction of the boron-phosphide monolayer. For structural optimizations, the Hellmann–Feynman forces using the conjugate gradient algorithm were calculated within a force convergence of 0.01 eV/Å [31]. The screened hybrid density functional HSE06 was employed to calculate the electronic band structures [32], which was confirmed as an accurate measure of band gaps for semiconductors. Herein, four types of defects were considered: B_v, P_v, D_v, and S-W defects. Within an orthogonal 4 × 3 supercell, the defects of B_v, P_v, and D_v can be constructed through removing one B atom, one P atom, and a pair of adjacent B and P atoms, respectively. The S-W defect was obtained by rotating any one B-P bond by 90°.

## 3. Results

### 3.1. Structural and Electronic Properties of Pure Boron-Phosphide Monolayer

**Figure 1:**
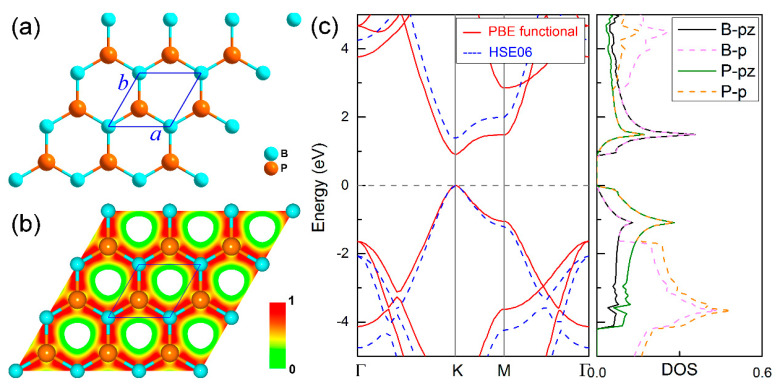
(**a**) Fully optimized structure, (**b**) charge density, and (**c**) electronic band structures obtained using PBE and HSE06 functionals and density of state (DOS) for boron-phosphide monolayer. Light blue and orange balls represent B and P atoms, respectively.

The structural and electronic properties of pure boron-phosphide monolayer are investigated firstly by comparing them with the defective systems. The optimized structure is shown in Figure 1a, which is composed of a pair of B and P atoms within a hexagonal primitive cell, exhibiting a space group of P-6m2. Similar to graphene, the boron-phosphide monolayer prefers to sustain in a planar sheet. Its optimized lattice constants *a* = *b* = 3.21 Å, and the B-P bond length is 1.86 Å, which are well consistent with previous studies [13,14,15]. In view of the charge density shown in Figure 1b, strong chemical bonds are formed between the B and P atoms, resulting in good thermal stability of the boron-phosphide monolayer [15]. Bader charge analysis reveals that such strong chemical bonds are mainly attributed to ion interaction with 0.77 electrons transferred from each B to neighbor P atoms (see Table 1). The electronic band structure and density of states (DOSs) are calculated and presented in Figure 1c. It can be seen that the boron-phosphide monolayer is a direct band gap semiconductor. Both the valence band maximum (VBM) and the conduction band minimum (CBM) appear at the K point in the reciprocal space. Its direct band gap of 0.91 eV is very close to the previous value [12,13,14,15]. By using the screened hybrid density functional HSE06, a larger band gap of 1.36 eV is obtained. At both the VBM and CBM, the interactions of π and π * bonds formed by the *p_z_* orbitals of the B and P atoms are critical to the direct band gap feature. For the *p_x_*, *p_y_*, and *s* orbitals, a strong σ bond is formed between the B and P atoms, which is located in the deep energy level and plays a key role in maintaining the structural stability of the boron-phosphide monolayer.

### 3.2. Structural, Elastic, and Electronic Properties of Defective Boron-Phosphide Monolayer

**Figure 2:**
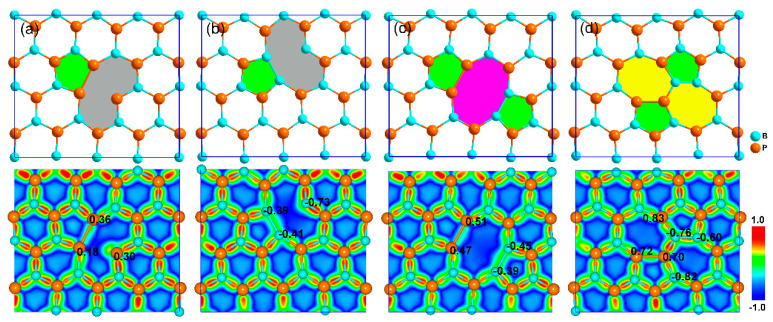
Optimized geometrical structures (up panel) and difference of charge densities (down panel) for defective boron-phosphide monolayers: (**a**) B_v, (**b**) P_v, (**c**) D_v, and (**d**) S-W defects. The value presented in the charge density is the Bader charge amount (unit in *e*) for the corresponding atom around the defect. Positive and negative values represent gaining and losing electrons.

The optimized geometrical structures of defective boron-phosphide monolayers are shown in Figure 2. Structural parameters and Bader charge amount transferred from the B to P atom around the defects are summarized in Table 1. It can be seen that all defective monolayers exhibit local reconstructions at the defects. As shown in Figure 2a,b, both the B_v and P_v defects can induce a five-membered ring and an irregular nine-membered ring because of the formation of P-P and B-B chemical bonds. Within the nine-membered rings, there is one P atom and one B atom forming double coordinated chemical bonds, which is different from the three coordinations in the pure monolayer. Viewed from the difference of charge density, these new formed P-P and B-B bonds around the B_v and P_v defects are relatively weak. Only small amounts of electrons are gathered at these chemical bonds, displaying much larger P-P and B-B bond lengths than those in phosphorene (*l*_P-P_ = 2.22~2.26 Å) [33,34] and borophene (*l*_B-B_ = 1.61~1.87 Å) [35,36]. Bader charge analysis reveals that the average amounts of electrons gaining/losing for the P/B atom around the defects are obviously smaller than 0.77*e* in the pure monolayer, indicating weak ion bond interactions between the B and P atoms. For the case of the D_v defect displayed in Figure 2c, a local 5-8-5-membered ring is formed. Similar to the B_v and P_v defects, the D_v can also result in the formation of weak P-P and B-B bonds. The B-P bonds around the D_v defect are weaker than that of the pure monolayer. However, the effects of the S-W defect are different, which can induce a 5-7-7-5-membered ring (see Figure 2d). In this case, the new formed P-P and B-B bonds are much stronger than the corresponding ones in the B_v, P_v and D_v defects, demonstrating much smaller bond lengths (see Table 1). The average B-P bond length around the S-W defect is very close to that of the pure boron-phosphide monolayer; so is the average amount of charge transfer, since the introduction of the S-W defect does not alter the area density but slightly changes the local structure. Thereby, a small local strain is induced. However, the B_v, P_v, and D_v defects can give rise to larger in-plane strains around the defects because of the reducing area densities, which can weaken the chemical interactions between the B and P atoms. Similar structural reconstructions induced by the defects have been observed in graphene [20].

**Figure 3:**
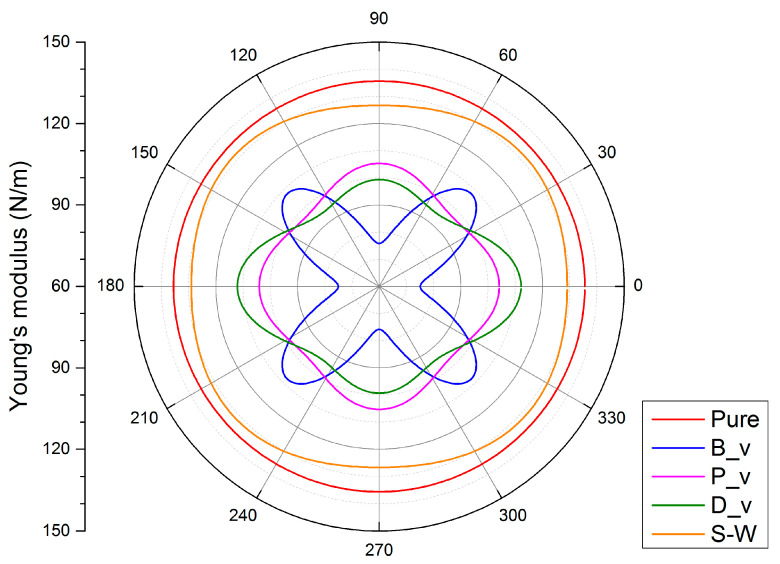
Polar diagrams of the in-plane Young’s modulus for pure and defective boron-phosphide monolayers.

To assess the structural stability of these defective monolayers, the formation energy is calculated as $E_{f}=E_{defect}-(E_{pure}-\mu_{removed})$, where $E_{defect}$ and $E_{pure}$ are the total energies of the defective and pure boron-phosphide monolayers, respectively. $\mu_{removed}$ is the chemical potential of the removed atom. Here, the chemical potentials for P and B atoms are obtained from 2D phosphorene and borophene. The calculated formation energy is summarized in Table 1. For the B_v and P_v defects, their formation energies are 4.54 and 6.25 eV, which are smaller than 6.64 eV of the D_v and larger than 3.76 eV of the S-W. This implies that the S-W is much easier to form, whereas the D_v is more difficult. In addition, the elastic property affected by the defects is also explored. Based on the calculated results of 2D linear elastic constants, including *C*_11_, *C*_22_, *C*_12_, and *C*_44_, in-plane Young’s modulus along an arbitrary direction *θ* (*θ* is the angle relative to the positive *x*-direction of the sheet) can be expressed as [37]

$$C(\theta )=\frac{C_{11}C_{22}-C_{12}{}^{2}}{C_{11}s^{4}+C_{22}c^{4}+\left(\frac{C_{11}C_{22}-C_{12}{}^{2}}{C_{44}}-2C_{12}\right)c^{2}s^{2}},$$

where *c* = cos(*θ*) and *s* = sin(*θ*). The polar diagrams of *C*(*θ*) for pure and defective boron-phosphide monolayers are displayed in Figure 3. It is clear to see that the modulus of defective monolayers is smaller than that of the pure monolayer, implying that the defective systems are softer than the pure ones. Moreover, the Young’s modulus of the pure layer is isotropic with a modulus of 135.5 N/m. However, the defective layers, including B_v, P_v, and D_v defects, exhibit anisotropic Young’s modulus. Such anisotropic feature is not obvious in the S-W defective layer because of its modulus range of 126.7~131.8 N/m. In summary, the defects can not only result in low in-plane modulus but also induce anisotropic elastic properties in the boron-phosphide monolayer.

**Figure 4:**
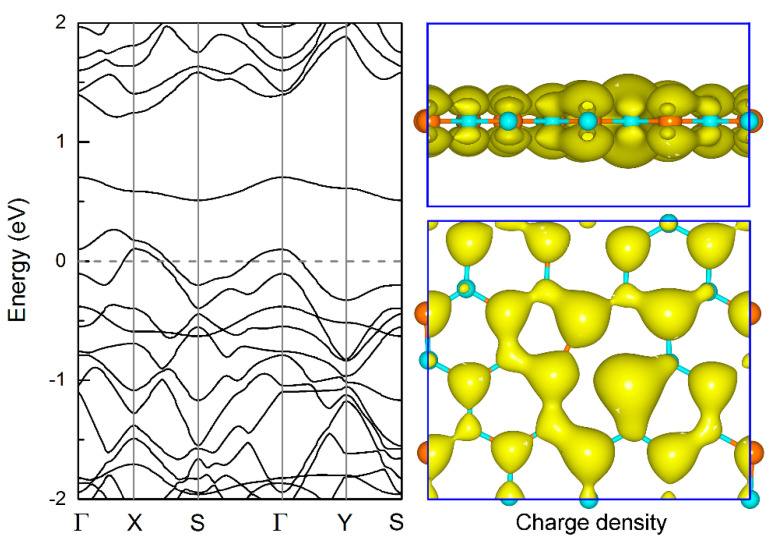
Electronic band structure and spatial charge density of the B_v defective boron-phosphide monolayer. The isosurface of charge density is set to 0.02 *e*·Å^−3^.

**Figure 5:**
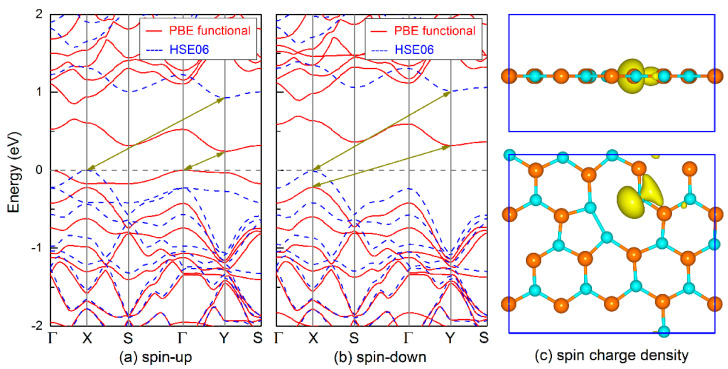
Electronic band structures for (**a**) spin-up and (**b**) spin-down channels, and (**c**) spin-charge density of the P_v defective monolayer. The isosurface of charge density is set to 0.02 *e*·Å^−3^.

Next, we calculate the electronic property of the boron-phosphide monolayer tuned by the B_v, P_v, D_v, and S-W defects. Figure 4 shows the electronic band structure and spatial charge density at the Fermi level for the B_v defective layer. Different from the semiconducting property of the pure boron-phosphide monolayer, the B_v system exhibits metallic with two bands crossing the Fermi level. By analyzing from the charge density, it can be seen that the Fermi level is mainly occupied by the *p_z_* orbital of P atom, and a small part comes from the *p_z_* orbital of B atom, forming the hybrid interaction of π bond between the B and P atoms. In addition, these *p_z_* orbitals are the main carriers and spread nearly in the whole surface areas, exhibiting good electronic conductivity. As shown in Figure 5a,b, the P_v system is a magnetic semiconductor with a total magnetic moment of 1 μ_B_. Both the spin-up and spin-down channels exhibit indirect band gap features. The corresponding band gaps are 0.24 and 0.54 eV predicted using PBE functional. By employing the screened hybrid density functional HSE06, much larger band gaps of 0.93 and 1.03 eV are obtained for the spin-up and spin-down channels, respectively. Here, it should be pointed out that the HSE06 functional has a great correction on the spin-up band structure, not only on the band gaps. For example, the PBE functional predicts that the VBM of the spin-up band structure is located at the Γ point, whereas the HSE06 functional reveals it is located at the X point. Although there are some differences in the band structure calculations, it does not affect the indirect band gap semiconducting properties and the total magnetic moment. In view of spin-charge density shown in Figure 5c, it can be found that the magnetism of the P_v monolayer is mainly attributed to the localization of the *p_x_* and *p_y_* orbitals for the double coordinated B atom. The small contribution comes from the *p_x_* and *p_y_* orbitals of the adjacent two P atoms.

**Figure 6:**
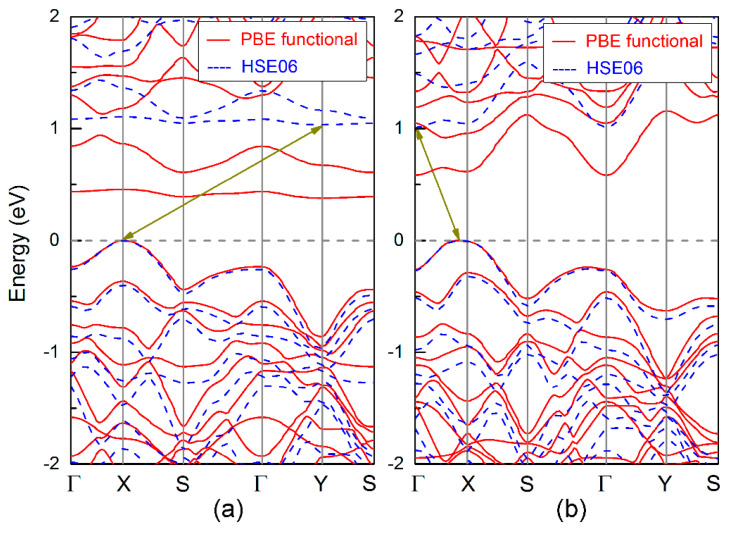
Electronic band structure for the defective boron-phosphide monolayers: (**a**) D_v and (**b**) SW defects.

Figure 6 shows the calculated band structures of the D_v and S-W defective boron-phosphide monolayers. One can see that both defective layers are indirect band gap semiconductors, which is different from the direct band gap of the pure boron-phosphide monolayer. For the D_v system (see Figure 6a), the VBM is at the X point, while the CBM occurs at the Y point, forming an indirect band gap between the X and Y points. The band gaps are 0.39 and 1.03 eV, predicted by using the PBE and HSE06 functionals. For the S-W defect layer displayed in Figure 6b, its VBM appears near the X point, and the CBM is located at the Γ point. An indirect band gap of 0.59 eV is obtained using the PBE functional. A larger indirect band gap of 1.01 eV is predicted by using the HSE06. As is well known, the DFT calculations underestimate the band gaps of semiconductors. However, the screened hybrid density functional HSE06 can predict a comparable band gap to the experimental value [32]. In addition, both the D_v and S-W defects can lead to direct-to-indirect band gap transition in the boron-phosphide monolayer. Similar band gap transition can also be induced in phosphorene by F- and I-doping [34]. Compared with the pure boron-phosphide monolayer, there is a common characteristic between the D_v and S-W defects; that is, without changing the three coordinated bonding characteristics for each atom, these two defects locally destroy the hexagonal symmetry of π bond interaction, which may be the reason for the indirect band gap semiconducting of the boron-phosphide monolayer rather than the orthogonal symmetry of the defect position. In order to verify this, we adopted a hexagonal 4 × 4 supercell to explore the effects of D_v and S-W defects on the electronic band structure of the boron-phosphide monolayer. The same results of indirect band gap features for these two defective systems indicate that the indirect band gap feature is independent of the symmetry of the defect position. Therefore, it can be concluded that the vacancy and S-W defects have significant regulations on the band structures of the boron-phosphide monolayer. The P_v, D_v, and S-W can result in the direct-to-indirect band gap transition, and the indirect band gaps are ~1.0 eV, which is comparable to that of silicon, suggesting the potential applications of the boron-phosphide monolayer in nanoelectronic devices.

### 3.3. Strain Engineering of Band Gaps for Pure Boron-Phosphide Monolayer

**Figure 7:**
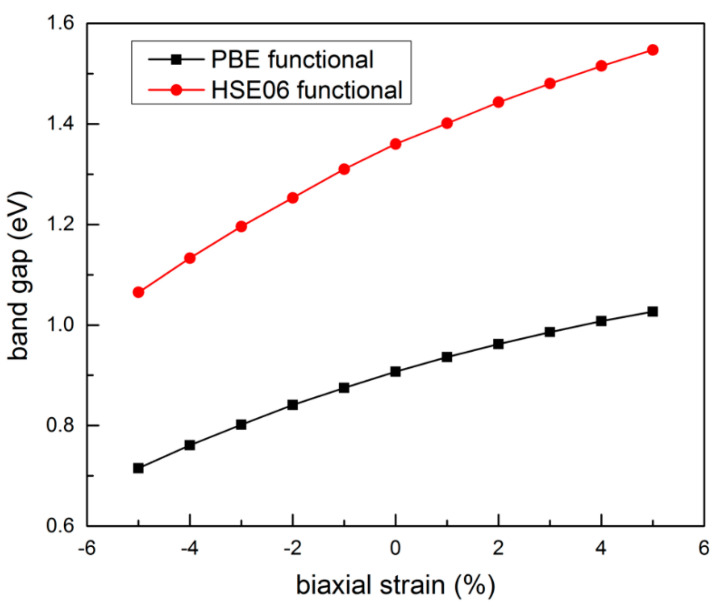
Band gap of pure boron-phosphide monolayer as a function of biaxial strain.

For 2D nanostructures, the strain plays a key role in their band gap engineering. Previous studies have confirmed that the in-plane strain is very effective for regulating the electronic band gaps of 2D monolayers. Herein, the in-plane strain is defined according to the following formula: *ε* = (*a* − *a*_0_)/*a*_0_, where *a* and *a*_0_ are the lattice constants for strained and equilibrium states, respectively. Figure 7 shows the calculated band gap of boron-phosphide monolayer as a function of the in-plane biaxial strain *ε*. In the range of −5 to 5%, both the PBE and HSE06 functionals predict a similar tendency of band gap. With the increasing tension strain, the band gap increases, while it decreases as the compression strain increases. The results of HSE06 functional calculations display that the band gap can be tuned from 1.07 to 1.55 eV in the range of *ε* = −5~5% with an average slope of 0.05 eV per strain, offering an effective method to tune the band gap of the boron-phosphide monolayer.

## 4. Conclusions

In summary, spin-polarized density functional theory calculations are employed to explore the effects of B_v, P_v, D_v, and S-W defects and in-plane biaxial strain on the structures and electronic properties of the boron-phosphide monolayer. The main results are as follows: (a) All of the defects can induce local atomic reconstructions in the boron-phosphide monolayer. (b) All of the defects can not only result in low in-plane Young’s modulus but also induce anisotropic elastic properties in the boron-phosphide monolayer. (c) Their effects on electronic properties are significant. The B_v defect can result in metallic property; the P_vacancy defect leads to magnetic semiconducting with indirect band gaps for spin-up and down-down channels. The D_v and S-W defects can induce indirect band gap semiconducting. The band gaps of these defective monolayers are ~ 1.0 eV, which is comparable to that of silicon. (d) The in-plane biaxial strain can monotonically tune the band gap of the boron-phosphide monolayer with an average slope of 0.05 eV per strain. The band gap increases with the increasing tension strain, while it decreases as the compression strain increases. The above results suggest that defects and in-plane strain are effective in adjusting the electronic properties of the boron-phosphide monolayer, which can promote further research and development in electronics and optoelectronics based on the boron-phosphide monolayer.

## Figures and Tables

**Table 1:** Properties of the pure and defective boron-phosphide monolayers: average bond length (Å), Bader charge amount (average number of electrons transferred from B to P atom around the defects, unit in *e*), formation energy *E_f_* (eV), in-plane Young’s modulus *C* (N/m), and electronic band gap *E_g_* (eV). The letters D and I in the brackets represent direct and indirect band gaps, respectively.

Systems	*l*_B-P_	*l*_B-B_	*l*_P-P_	Δ*Q*	*E_f_*	*C*	*E_g_*
pure	1.86	—	—	0.77	—	135.5	1.36 (D)
B_v	1.90	—	2.46	0.28	4.54	75.0–108.0	metal
P_v	1.90	2.12	—	0.51	6.25	97.1–104.1	0.93 (I, spin-up) 1.03 (I, spin-down)
D_v	1.91	2.06	2.47	0.49	6.64	94.2–112.1	1.03 (I)
S-W	1.85	1.70	2.16	0.75	3.76	126.7–131.8	1.01 (I)

## Data Availability

The data is available on reasonable request from the corresponding author.
